# Bridging Nanomanufacturing and Artificial Intelligence—A Comprehensive Review

**DOI:** 10.3390/ma17071621

**Published:** 2024-04-02

**Authors:** Mutha Nandipati, Olukayode Fatoki, Salil Desai

**Affiliations:** 1 Department of Industrial and Systems Engineering, North Carolina Agricultural and Technical State University, Greensboro, NC 27411, USA; mrnandipati@aggies.ncat.edu (M.N.); omfatoki@aggies.ncat.edu (O.F.); 2 Center of Excellence in Product Design and Advanced Manufacturing, North Carolina Agricultural and Technical State University, Greensboro, NC 27411, USA

**Keywords:** artificial intelligence, digital manufacturing, nanocharacterization, nanomaterials, nanomanufacturing

## Abstract

Nanomanufacturing and digital manufacturing (DM) are defining the forefront of the fourth industrial revolution—Industry 4.0—as enabling technologies for the processing of materials spanning several length scales. This review delineates the evolution of nanomaterials and nanomanufacturing in the digital age for applications in medicine, robotics, sensory technology, semiconductors, and consumer electronics. The incorporation of artificial intelligence (AI) tools to explore nanomaterial synthesis, optimize nanomanufacturing processes, and aid high-fidelity nanoscale characterization is discussed. This paper elaborates on different machine-learning and deep-learning algorithms for analyzing nanoscale images, designing nanomaterials, and nano quality assurance. The challenges associated with the application of machine- and deep-learning models to achieve robust and accurate predictions are outlined. The prospects of incorporating sophisticated AI algorithms such as reinforced learning, explainable artificial intelligence (XAI), big data analytics for material synthesis, manufacturing process innovation, and nanosystem integration are discussed.

## 1. Nanoscale Science and Technology

Nanotechnology refers to technologies that are applied at the nanometer level and have applications in real life [1]. These technologies involve controlling and manipulating matter and operating in dimensions from 1 to 100 nm. It can be further streamlined as the design, characterization, manufacture, and shape- and size-controlled application of matter whose smallest functional organization is on the scale of one billionth of a meter to make matter, systems, and devices with fundamentally novel properties and functions [1,2,3,4,5,6]. Nanotechnology has resulted in breakthrough solutions in many fields and real-life applications, such as smart materials, nanomanufacturing, electronics, water, drug delivery, and national security. The pivotal components of nanotechnology include nanomaterials, nanomanufacturing, and the application of various systems, such as physical, chemical, and biological, to create new materials and systems at nanoscale with enhanced properties [3,4,5]. The subsequent sections provide an in-depth understanding of nanomaterials, nanomanufacturing, digital manufacturing, and artificial intelligence. They set the groundwork on each topic individually, before delving into exploring the intricate relationship between artificial intelligence and nanomanufacturing.

### 1.1. Nanomaterials

Nanomaterials are the key elements of nanomanufacturing, with dimensions of one billionth of a meter [7]. According to author Vollath, nanomaterials possess physicochemical properties that differ from the parent material based on their size and shape. They also exhibit a variety of geometric configurations classified into zero-dimensional (nanoparticles), one-dimensional (nanorods), two-dimensional (films, layers, and sheets), and three-dimensional (nanocomposites, core shells, multi nanolayers, bundles of nanowires and tubes) [8]. Nanomaterials are categorized according to their composition, with distinctions based on form, size, characteristics, and constituents, resulting in polymeric, carbon-based, metal oxides, semiconductor nanomaterials, and lipid-based variants. Among the widely employed nanomaterials, silver, carbon-based materials, and tungsten oxide are prevalent choices [9]. Nano-silver is used for antimicrobial applications due to its physicochemical and biological properties [10]. Carbon-based nanomaterials, with their extraordinary mechanical and electrical properties, are widely used in battery technologies [5,6,7]. Nano ceramics are used in wear-resistant coatings due to their higher hardness, toughness, and wear-resistance properties [11].

### 1.2. Nanomanufacturing

**Figure 1:**
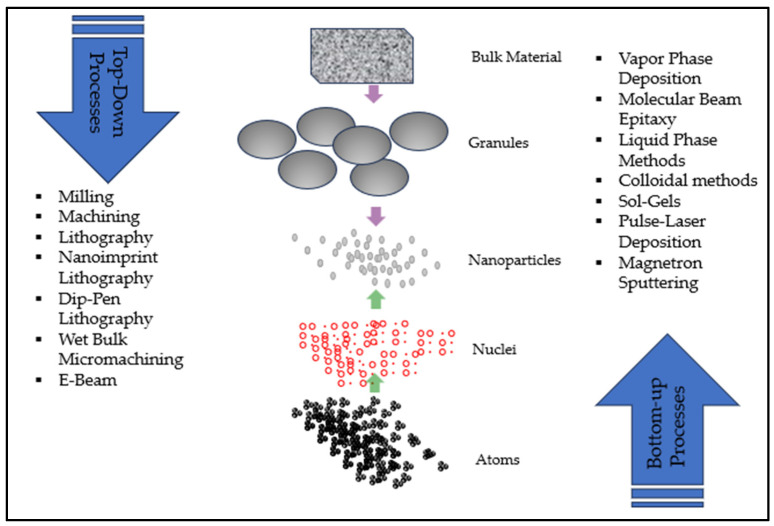
Production of nanomaterials (top-down and bottom-up processes).

Nanomanufacturing denotes the production processes of fabricating nanomaterials, structures, devices, and systems at one billionth of a meter scale, utilizing either a top-down or a bottom-up method [12,13], as shown in Figure 1. Top-down approaches start with a macroscopic material and incorporate their way down to smaller-scale details. Some of the most widely used top-down processes are milling, machining, lithography, and micromachining. In milling, a suitable powder charge is placed in a high-energy mill, along with a suitable milling medium. This is to reduce the particle size and blending of particles in new phases [1]. Machining includes material removal at the nanometric level, conventional cutting, grinding, and polishing [2]. Top-down lithography is suitable to fabricate fine, mass-producible patterns. Nanoimprint lithography is a method of transferring a pattern shape of a mold on the target materials by contacting a mold. In dip-pen lithography, high-resolution nanopatterns are obtained by delivering materials via capillary transport using scanning probe microscope tips [3]. Bulk micromachining is a prevalent technique utilized in the creation of a vast range of microstructures, varying in complexity, within silicon wafers. This procedure predominantly incorporates wet bulk micromachining, which in turn employs wet etching. Wet anisotropic etching serves as the central process in the fabrication of a myriad of components, all uniquely shaped, being used extensively within microelectromechanical system (MEMS) applications [4]. Electron beam lithography (E-Beam) uses an electron beam at a voltage of approximately 15 kV and is a fabrication of nanopatterns of conductive thin films down to 100 nm [5]. Bottom-up techniques, on the other hand, begin by creating and synthesizing the desired molecules using various nanomanufacturing processes, such as self-assembly, vapor phase deposition, liquid-phase methods, pulse-laser deposition, and molecular beam epitaxy [1,12,13,14,15]. Gas-phase synthesis and liquid-phase formation are the two basic methods in bottom-up processes. Some of these widely employed methods are vapor phase deposition, molecular beam epitaxy, colloidal methods, sol–gel, pulse-laser deposition, and magnetron sputtering [6]. In vapor phase deposition, a substrate is exposed to one or more volatile precursors, which then decompose the substrate and react with it to produce the desired deposit. Molecular beam epitaxy works on vacuum evaporation, where direct impingement of thermal molecular and atomic beams takes place under ultra-high vacuum conditions. In the sol–gel process, metal oxides are fabricated from small solid molecules [16]. The deposition in pulse-laser technology involves focusing a pulsed laser beam on the source material so that it deposits on a substrate inside a high vacuum chamber. Another thin-film deposition method in use, known as magnetron sputtering, is a process where atoms are ejected from a target by energetic ions [17].

Different nanomanufacturing processes are in practice in the manufacturing industry, and the top five nanoscale manufacturing processes [18], according to the American Society of Mechanical Engineers (ASME), are printing of 3D solid materials, photolithography, microscale assembly, 3D printed memory polymers, and nanomanufacturing of polymer 2D materials. The principle behind the process of printing integrated 3D solid materials is via multiple layering of melted polymers or metals [19,20]. Photolithography is a process where the optimal mask is subjected to an ultraviolet (UV) light to facilitate the transfer of geometric pattern from the mask to a substrate [21]. Assembling components across multiple-size scales is the process behind the microscale assembly of nanostructured metamaterials. Modulated surface energy is used in the adhesion of flexible tools to accommodate assembly [22]. In the process of 3D-printed customized shape memory polymers, small and lightweight actuator systems are 3D printed with the material’s response to its environment in contrast to the physical integration of mechanical components [23,24,25]. In the scalable nanomanufacturing of 2D polymer materials, it employs the principle of photolithography to generate high-density small patterns on polymers [26,27].

### 1.3. Digital Manufacturing

According to author Li, conventional nanomanufacturing processes aim towards enhancing the quality and purity of particles, which has enormous potential for optimization [28]. Manufacturing firms across sectors encounter tremendous problems in effectively designing and managing modern products [29]. To address the intricacies in the development of modern products, digital product development is utilized, along with the fusion of technologies to build cyber-physical systems (CPSs) and smart factories [30].

As the era of industry 4.0 continues to broaden, organizations are driven to diversify their products into various nanoscales, resulting in the adoption of more efficient and scalable production methods [31]. CPSs are crucial in various domains, like robotics, defense systems, and electric infrastructure, due to security threats [32]. Industries are transitioning to digital manufacturing from the age-old traditional types, as stipulated by Industry 4.0. The relationship between nanomanufacturing and digital manufacturing is depicted in Table 1.

### 1.4. Artificial Intelligence

Artificial intelligence (AI) is a pervasive subdivision of computer science that chiefly emphasizes the development of intelligent machines competent in executing tasks that conventionally necessitate human intelligence, as illustrated by Wang et al. [34]. It is an inherently data-dependent technology that focuses on automatic learning as well as predictive capabilities. AI shows excellence in carrying out tasks in an unsupervised manner and avoids human intervention in decision-making. It also has greater fitness ability with functions and fast computing speed. AI has proved its superiority in various fields, such as autonomous driving [35], natural language processing [36], medical diagnosis [37], computer vision [38], intelligent manufacturing [39], computer (imaging) vision [40], spectral informatics [41], ultrafast photonics [42], fiber lasers [43], and drug delivery [44]. AI is increasingly being integrated into various nanomanufacturing processes, such as nano assembly, the creation of nanotubes, nanomedicine, nanorobotics, and nanosensors, denoting its essential role in the advancement of nanotechnology [45,46,47,48,49,50,51]. Table 2 shows an exhaustive list of such nanomanufacturing processes that incorporate the use of AI.

**Figure 2:**
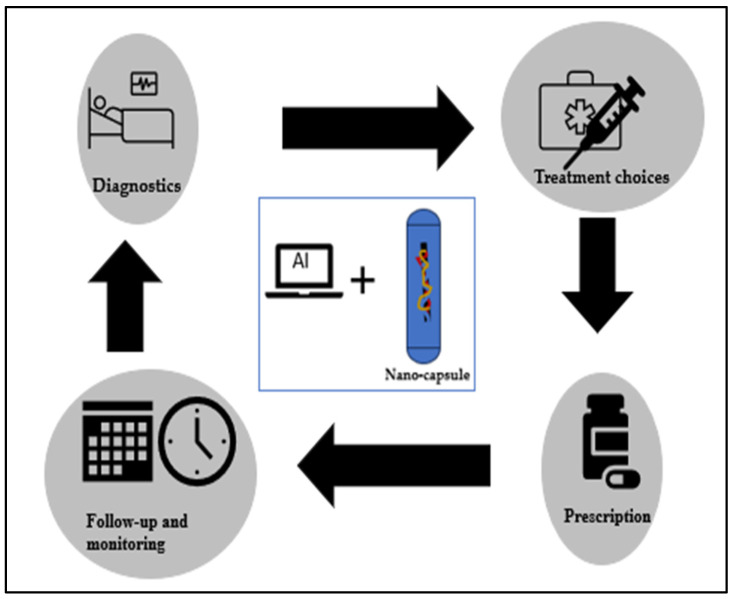
Nanofabricated drug delivery capsule application with AI.

Authors Varahramyan and Lvov discussed how nanomanufacturing methods are saving human lives through nanoengineered capsules for sustained release drug delivery [62]. These nanoengineered capsules can provide cancer treatment through the integration of AI, as proposed by Adir et al. [54]. The mechanical, electrochemical, and spectral properties of nanosensors enhance the diagnostic profile for cancer patients, allowing them to have early detection, a treatment plan, and monitoring of the disease. As shown in Figure 2, during the diagnostic stage, nanosensors can detect extremely low concentrations of cancer biomarkers [63,64]. Integration of AI algorithms in the data analysis can allow recognition of combinatory treatments. Prescriptions are prepared with nanomedicine (nano-capsules), which control drug dosing by adjusting the drug release rate based on the patient’s diagnosis and the treatments identified. Nanoparticle-based image analysis using convolutional neural networks has been widely used during the monitoring stage, and follow-up is conducted based on the AI model’s outcome.

### 1.5. Interdependency of Nanomaterials, Nanomanufacturing, Digital Manufacturing, and Artificial Intelligence

**Figure 3:**
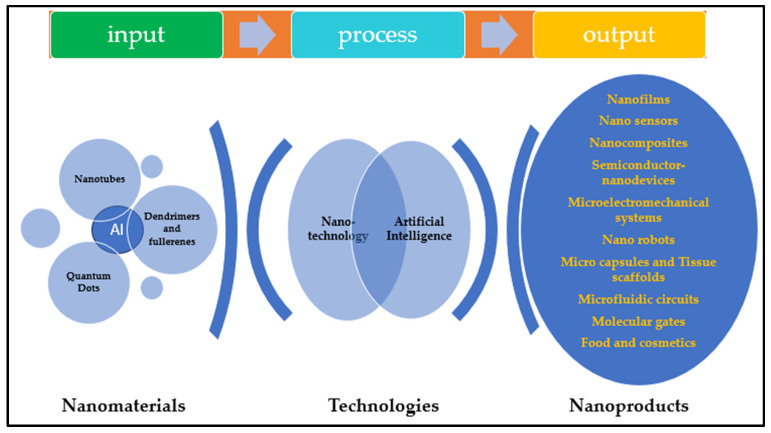
Alignment of artificial intelligence in the nanomanufacturing process.

Nanomaterials, nanomanufacturing, digital manufacturing, and artificial intelligence are pivotal elements in industry 4.0. The application of predictive algorithms has resulted in novel paths in material discovery and optimization. Digital manufacturing, which centers on digital design and control of the manufacturing processes, emerges as a promising approach to addressing challenges in complex geometries, material optimization, and nanoscale material design [65]. According to Dopico et al. [66], this approach integrates information, computation, sensors, software, networking, and automation to carry out assigned tasks. The combination of AI and DM drives nanomanufacturing processes, streamlining the flow from inputs known as data. These data can be extracted from nano/micro-scale designs, CAD inputs, manuals, or published technical journals and give desired outputs known as outcomes [67,68,69,70,71,72,73]. Artificial intelligence proves instrumental in enhancing production processes, predicting novel nanostructured materials, improving efficiency and ensuring product quality (predictive maintenance) and supply chain optimization [74]. Figure 3 exemplifies a manufacturing process where input materials such as nanotubes, quantum dots, and dendrimers are utilized to produce outputs such as nanofilms, nanosensors, and nanorobots.

Optimally integrating artificial intelligence (AI) within nanomanufacturing, in line with the benchmarks set by Industry 4.0 and digital manufacturing, equips manufacturing companies with a competitive edge and the agility to meet the rapidly changing demands of the global market. Such coordination also reveals opportunities for comprehensive optimization of production processes, real-time monitoring capabilities, and the freedom to undertake research and development. The ensuing segment of this manuscript delves deeper into the specifics of utilizing AI in the realm of nanomanufacturing.

## 2. Artificial Intelligence in Nanomanufacturing

In the field of exploring nanomanufacturing, AI is effective in predicting the properties and behavior of nanomaterials. Additionally, AI algorithms have been employed to optimize various aspects of nanomanufacturing processes, such as improving efficiency and reducing defects. the conversion of nanomaterials into final products strategically leverages artificial intelligence to achieve precision, minimize wastage, and optimize efficiency [74]. Support vector machines (SVMs) and artificial neural networks (ANNs) serve as pivotal tools to find the relationships between raw materials and the nano products produced. The application of AI extends beyond relationship exploration; it underscores its instrumental role in material synthesis and nanocharacterization.

### 2.1. Material Synthesis

Nanoparticles, owing to their distinctive properties, find extensive applications in fields such as medicine, material science, chemistry, and optoelectronic sensing. The synthesis of nanoparticles involves navigating an extensive array of synthetic paths, resulting in challenges in achieving precise control over particle sizes and the desired properties of the synthesized nanoparticles. As expressed by Chen et al., optimizing this synthesis becomes complex, requiring consideration of multiple parameters [75]. In addressing these challenges, Mijwel et al. [76] explored the application of AI, specifically employing feedforward neural network (FFNN) and recurrent neural network (RNN) models to replicate the adsorption capability of functionalized carbon nanotubes (CNTs) for methylene blue removal. It was further revealed that increasing the number of neurons in a neural network is a critical parameter that influences the model’s capacity to learn and represent complex relationships in the context of material synthesis. The results revealed exemplary performance, with correlation coefficients of 0.9471 and 0.9658. Water plays an integral role in material synthesis, particularly in water treatment modeling, as emphasized by Safeer et al. [77]. Methylene blue (MB), a synthetic dye with a deep blue color, has an array of applications in the synthesis of nanoparticles because it is utilized for the fabrication, imaging, and modification of nanoparticles. For safe disposal and improving water quality assessment, neural networks are employed for water treatment modeling [77]. Several neural networks have also been applied in the synthesis, characterization, parameter optimization of nanocrystals, detection, and measurement of nanocantilever positions, fabrication, and post-fabrication imaging of structures via focused ion beam milling (FIB) [78,79,80].

These structures are extensively applied in the nanophotonic, seeded growth of nanostructures, and many other applications [81,82,83,84,85]. In addition, several machine-learning algorithms have been employed to assist in the synthesis of nanoparticles, as shown in Table 3.

#### Nanocomposites

Nanocomposites, heterogeneous materials with structures, components, or phases ranging from 1 to 100 nm, find applications in diverse fields, including food, biomedical [96,97,98,99], and electroanalysis [26,100,101,102]. They offer a unique approach for enhancing properties through the integration of nanoscale reinforcements developed through material synthesis. In the work by Mazahery et al. [103], a machine-learning algorithm was proposed as an alternative to costly experimental investigations in various manufacturing areas. The study employed artificial neural network (ANN) models for the numerical modeling of metal composites reinforced with nano-sized ceramic particulates. This approach, combining the finite element method (FEM) and ANN algorithms, accurately predicted wear behavior. The Levenberg–Newton method algorithm trained an ANN with a 1-hidden-layer, 8-neuron architecture. In the research by Harsha et al. [104], ANN models were developed to predict the tensile strength of Al-Al_2_O_3_ nanocomposites fabricated through powder metallurgy. Achieving a 98% accuracy with a 5-5 hidden layer architecture, the study highlighted the significant impact of the number of neurons and hidden layers on ANN model efficacy. Unlike Mazahery et al. [103], it was evident that ANN models vary with problem-specific parameters. Leveraging machine-learning models enables researchers to gain insights into the integral role of material synthesis and nanomaterials in the fabrication and customization of nanocomposite materials. Table 4 succinctly outlines studies utilizing ML models for predicting the properties of polymeric nanocomposites.

### 2.2. Nanomanufacturing Process

Nanomanufacturing forms the foundation for almost all areas of nanomaterial research and development, specifically focusing on the utilization of these materials for sophisticated, versatile devices across diverse technological sectors. These sectors include electronics, energy systems, medicine, and health care [117,118,119,120,121]. Techniques for creating nanostructured materials and nanoscale structures are categorized into top-down and bottom-up methods. The top-down approach applies lithography techniques to pattern nanoscale structures, often used in electronics industries, whereas the bottom-up method assembles discrete nanoscales structures through molecular or colloidal interactions in two and three dimensions [117,122]. The integration of AI has proven its superiority across diverse fields, one of which is the semiconductor industry.

#### Lithography–AI Case Study

The significance of lithography in the semiconductor industry and the increasing use of machine-learning algorithms, particularly deep learning, is applied in lithography simulation tasks. Lithography involves transferring a designed mask pattern onto a wafer, and it is composed of two stages: optical and resist simulation [123]. Due to high cost and time-consuming experimental verification, lithography simulation for process development and performance verification has been relied on. Various machine-learning algorithms are applied in lithography simulation for tasks like performance modeling, hotspot detection, and mask optimization. In performance modeling, Lin et al. [124] investigated performance-learning modeling under uncertainty and lithography modeling with active learning. Watanabe et al. [125] proposed a compact resist model using CNN for real-time chip simulation and achieved a 70% reduction in prediction errors compared to conventional models. Lin et al. [126] presented a novel approach to lithography simulation using transfer learning, in which residual neural networks (ResNet) were utilized for high-performance resist modeling that reduces the amount of data needed for accurate modeling. Other machine-learning-based techniques have been proposed to improve simulation quality and to predict height of resist after exposure in constructing accurate and efficient resist models [127]. Also, owing to the advancement in deep learning, other attempts have been made to solve the mask 3D effect problems using deep neural networks [128,129,130]. Tenable et al. [131] proposed an AI algorithm (CNN) to reduce the calculation time for diffracted waves from EUV masks. This CNN prediction is 5000 times faster than rigorous electromagnetic simulations. The authors further stated that the eigenvalue decomposition method can be used to accelerate the calculation. 

Nanoimprint lithography (NIL) is characterized as a high-resolution, throughput, and cost-effective technology for developing nanoscale features in a relatively short amount of time [132,133,134]. Achieving defect-free imprints requires meticulous control and optimization of process parameters. Akter and Desai [135] predicted defect-free imprints by utilizing ANN algorithms. These ANN models (GRNN, BPNN, PNN) were trained using several input factors and of these three models GRNN predicted an accuracy of 55% and 90% for stage 1 and stage 2, respectively.

### 2.3. Nanocharacterization

Material science has seen a gradual transition over the past decades from creating innovative materials using raw computational approaches to creating linked methods that increase the reliability of the results through experimental validation and computational predictions. Products manufactured at the nanoscale necessitate the use of extremely sensitive and highly precise instruments for appropriate measurement. This process, known as nanocharacterization, involves the detailed analysis and measurement of nanoscale materials and structures [136]. Additionally, machine learning (ML) has found applications in polymeric nanocomposites for material property prediction, microstructural characterization, process optimization, and quantification of uncertainties arising from complex production processes [137,138]. 

#### 2.3.1. AI in Nanocharacterization

Salah et al. [139] applied ML models to predict the absorption of CNT composites. In another study, Yusoff et al. [140] used ML and a multilayer perceptron network technique to improve process parameters and predict rheological properties such as the absorption index of nanosilica. Khanam et al. [141] utilized ANNs to optimize and predict the mechanical and chemical properties of graphene nanoplatelets nanocomposites. Other studies employed various computational techniques such as ANN, Bayesian inference, and other optimization algorithms to predict and analyze the material properties of hybrid nanocomposites [142], electrical conductivity of composites [137], and potential applications of CNT nanocomposites in damage detecting sensors [143]. 

Fatigue and creep are examples of some mechanical properties that are suitable for ANN analysis and ANFISs (adaptive neuro fuzzy inference systems) [144], while ANNs are suitable for predicting impact strength and yield strength. In addition, CNN has been applied to analyze images and make quantitative predictions about the mechanical behavior of composites by using the various microstructure grid sizes of the composites that are obtained from SEM analysis [105]. Several other studies have applied AI methods in the characterization of nanomaterials [145,146]. In other studies [147,148,149], the Levenberg–Marquardt gradient descent, Gauss–Newton algorithm, and genetic algorithms are commonly used for thin-film nanocharacterization.

#### 2.3.2. Hotspot Detection

Hotspot detection refers to the identification and analysis of areas on a semiconductor wafer or mask that are prone to manufacturing defects or variations. It is crucial for ensuring the reliability and performance of semiconductor products. Various data-mining and machine-learning techniques, including ANN, SVM, deep CNN, and GRNN, have been integrated into recent works to enable quick and precise hotspot detection. Nagase et al. [150] trained an ANN kernel directly using 2D hotspot image patterns, achieving approximately 55% hotspot detection accuracy. Ma et al. [151] developed data-mining algorithms for clustering 2D hotspot patterns, resulting in a library to automatically categorize hotspots. Ding et al. [152] proposed hotspot detection with critical feature extraction and classification using ANNs, achieving a small detection false alarm rate and an average detection accuracy of over 90%. Furthermore, Duo Ding et al. [153] used machine-learning algorithms to predict hotspots, employing deep CNN to precisely identify hotspots, outperforming other neural networks and fuzzy-matching techniques. Yang et al. [154] utilized deep CNN to identify false negative results, demonstrating superior performance compared to existing hotspot detectors in [155,156]. In addition, Shin and Lee [157] described a deep convolutional neural network approach for precisely identifying lithography hotspots in semiconductor layout designs, outperforming previous machine-learning and fuzzy-matching techniques with high detection rate and false alarm reduction. These studies have contributed to advancements in hotspot detection using machine-learning techniques, demonstrating improved accuracy and performance in semiconductor manufacturing. 

#### 2.3.3. Spectroscopy

Machine learning provides unprecedented opportunities in the analytical sciences for extracting data from complex datasets in fields such as spectroscopy, mass spectrometry, and NMR, among other fields. Particularly in techniques like Raman and surface-enhanced Raman scattering (SERS), extensive datasets of complex chemical mixtures’ vibrational spectra are collected for the analysis or imaging of chemical systems [158]. In addition, by leveraging the valuable information they provide about the chemical composition, structure, and surface characteristics of nanomaterials, researchers and manufacturers can gain a better understanding of the features and optimize their synthesis and production processes [159]. In nanomanufacturing, fabrication of SERS nanostructures can be achieved through either a top-down or a bottom-up approach. Among the different types of nanofabrication methods, lithographic techniques with electron beams [159,160,161] and focused ion beams and UV light have been greatly explored [162,163,164,165]. Bottom-up approaches have also been used to fabricate SER nanostructures via self-assembly, nanosphere lithography, and solution-based synthesis methods [166,167,168,169].

AI algorithms have highlighted the significant potential of SERS-based platforms for bio-analytical applications in the analysis of complex Raman spectra [159]. By utilizing spectrum data and AI-based algorithms, the accuracy of determining the findings of Raman spectra can be significantly improved. Important parameters in spectra preprocessing tasks are noise reduction, baseline correction, training, validation, normalization, and testing of the obtained raw datasets [170,171]. A kernel function is proposed for the optimal separation of the Raman spectrum to achieve more complex Raman spectral analysis in areas such as bacteria isolation [172]. Furthermore, the application of ANNs, deep learning, and machine learning has been seen in various tasks such as image recognition, photoinduced DNA damage [173], biomedical applications in cancer diagnosis, and detection of exosome proteins [174,175,176,177,178,179]. In NMR, 2D NMR reveals more information about the spectrum; however, the measuring times of 2D NMR increases astronomically with sampling numbers. In Kong et al. [180], a deep CNN was applied to speed up 2D nanoscale NMR spectroscopy, demonstrating enhanced sensitivity and accuracy in molecular structure analysis. The researchers demonstrated the effectiveness of the approach in pattern identification and noise cancellation, leading to enhanced sensitivity and accuracy in molecular structure analysis using NMR. Several authors have discussed how machine learning can be utilized with spectroscopic ellipsometry to also replace complex optical modeling [181,182]. Raman spectroscopy can also be used to identify known compounds and unknown materials and determine their number of layers [183,184,185,186].

In conclusion, the integration of machine learning (ML) with advanced spectroscopic techniques has revolutionized the field of nanomanufacturing. The ability to extract valuable insights from complex chemical mixtures’ vibrational spectra, particularly in the context of Raman and SERS techniques, has significantly advanced the characterization and optimization of nanomaterials and nanomanufacturing processes.

#### 2.3.4. Multilayer Device Measurement Methods

Electron microscopy, including critical-dimension scanning electron microscopy (SEM) and transmission electron microscopy (TEM), plays a crucial role in the analysis of multilayer thickness, defect analysis, and analyzing nanoscale structures [187,188,189,190,191,192]. In Kondo et al. [189], the authors revealed that ML can be applied to improve the imaging speed of SEM, allowing for the detection of faults or defects at high resolution using rapidly obtained low-quality images as inputs and slowly acquired high-resolution images as outputs. This allows researchers to visualize the distinct layers within a multilayer device and examine their composition, structure, and performance characterization. According to Choi et al. [193], either a multi-sample or a multi-angle can be used to increase the amount of information with regards to the measurement, and having samples of fixed optical constants as added information can result in improvements in the accuracy and uniqueness of thickness characterization when using these measurement devices. ML can save time during characterization, which results in substituting optical modeling [194,195]. 

AI plays an integral role in the nanomanufacturing process, particularly in material synthesis, lithography, hotspot detection, spectroscopy, and characterization methods. The use of AI in these areas has significantly transformed the way nanoparticles are synthesized and semiconductors are manufactured. AI models continue to evolve, becoming more sophisticated and efficient, enhancing the capabilities of nanomanufacturing with the applications of various machine-learning and deep-learning models, as detailed in the following section.

## 3. Machine Learning and Deep Learning in Nanomanufacturing

**Figure 4:**
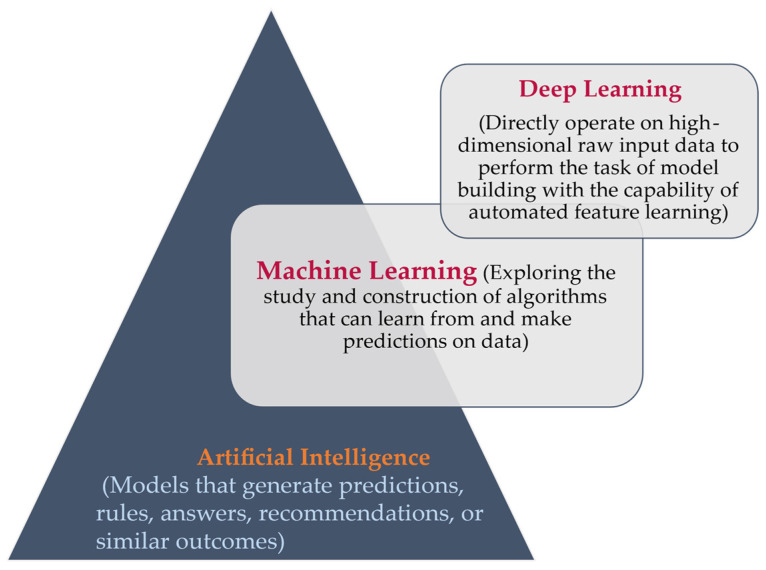
Machine learning and deep learning as AI tools.

As an emerging technology that primarily focuses on data-driven analysis and processing, AI has significantly contributed to the evolution of various manufacturing processes. In particular, the application of AI has brought about groundbreaking insights and advancements in the field of nanomanufacturing. One of the challenges in adopting AI into the nanomanufacturing process is the identification of appropriate machine-learning algorithms in the material’s design. Costly experiments and the complex calculations needed to conduct research on nanomaterials (NMs) and nanotechnology opened gates to advanced technologies like artificial intelligence. The various structures and properties of NMs are quickly revealed by machine-learning algorithms. It was highlighted that the huge data generated from thousands of papers published per year related to the structures, properties, adsorption properties, and catalytic behavior of NMs is not sufficiently used for NMs [196]. This research developed the quantitative structure–property relationship (QSPR) models and identified promising materials for specific applications with machine-learning algorithms. As mentioned by Jia. Y et.al [197], machine-learning algorithms are widely used in the exploration of new materials to study the defects and properties of nanomaterials. With various machine-learning algorithms in practice, researchers are often ambiguous about which algorithm to use. After reviewing various algorithms used by researchers, a consolidated table is made for easy reference, as shown in Table 5. All these algorithms are used in machine-learning and deep-learning algorithms, which are part of artificial intelligence. AI systems are designed to mimic human thinking and work autonomously. The Venn diagram given by Goodfellow et al. [198] shows the relation between AI, ML, and DL, and the same is used to show the relation between them with tasks performed by ML and DL, as shown in Figure 4. AI systems consist of machine-learning and deep-learning algorithms to predict the outcomes.

The AI algorithms used in nanomanufacturing are from four different categories, as shown in Table 6. These categories comprise supervised, unsupervised, reinforcement, deep, and hybrid learnings. A detailed understanding of each method is fundamental for effective application. In essence, supervised learning entails the training of models based on known input and output data, which consequently facilitates the prediction of future outputs. In unsupervised learning, algorithms aim to find patterns and structures in input data. The algorithms allow models to learn based on feedback from their actions in an environment in reinforcement learning. The final and most important one is deep learning, a sub-field of machine learning, which uses neural networks with multiple layers for complex pattern recognition. 

Implementing AI in nanomanufacturing is a complex process that presents various challenges and prospects for various reasons, such as precision requirements, quick and accurate detection of defects, data security [220,221], technological limitations, and sensitive predictive analysis, and this has made people think about the challenges and prospects of implementing AI, which are emphasized in the following section. 

## 4. Challenges and Prospects in Implementing AI in Nanomanufacturing

With AI adoption still at the initial stages in nanomanufacturing, it faces challenges in implementation, such as data availability, understandability, security, meeting regulatory requirements, etc. To mitigate all the challenges, researchers have proposed AI algorithms such as convolutional and deep neural networks, which are more powerful than traditional algorithms such as classifiers and regression models.

### 4.1. Challenges and Potential Solutions

Data availability: Despite the vast potential of nanotechnology across diverse domains, it is imperative to address the prevailing challenges concerning data assembly and alignment to facilitate the evolution of AI models [222]. For an AI model to function efficiently, it needs large quantities of quality data, which could be challenging to collect. The authors in papers [223,224] shed light on data-cleaning issues, specifically mentioning the deliberate or inadvertent inclusion of wrong data in training sets, which drastically affect the accuracy of the model and predictions. The authors also mentioned that the management of different versions of the datasets is a difficult task and errors that occur during this process are often hard to detect and correct. Data sparsity and quality are also considered as challenges due to missing data in the high-dimensional data. Noisy or incomplete data may pose a challenge in achieving the accuracy of machine-learning models [225].

Overcoming data availability challenges necessitates adopting a systematic approach towards data collection and cleansing, as endorsed by researchers. Additionally, they suggest employing data imputation strategies in managing absent data. This methodology enhances data input quality into AI models, thereby refining their precision and predictability [223,225]. For addressing the complexity and multi-scaled dimensions of nanomanufacturing data, the application of data fusion techniques has been proposed [224]. Furthermore, the establishment of public–private collaboration is advocated to stimulate data accessibility and dissemination [222].

Understandability: Results produced by AI and ML are often not clear (lack of transparency) and are sometimes like a black box. This could lead to a challenge while performing sensitive analysis during nano-characterization. A large concern in the implementation of AI is addressed in the paper by Von Eschenbach [226]: the lack of transparency and understandability in how AI algorithms make predictions. Wischmeyer’s paper [227] further emphasizes the black box issue. The author argues that the opacity of AI systems needs to be analyzed so that their predictions can be interpreted and understood. Carabantes’ paper [228] also stresses the importance of understandability in AI systems. It highlighted the issue of AI systems in decision-making, which can have a significant impact on the decision-making process. All these authors highlight the serious challenge of data understandability in AI. The authors emphasize the necessity for transparency to enable the effective regulation of AI.

To address the challenge of comprehending artificial intelligence (AI) models, the implementation of explainable AI (XAI) models has been suggested [226]. These models deliver an in-depth explanation for each decision they make, providing enhanced transparency and interpretability. This advantage is particularly useful in sensitive operations during nano-characterization. Additionally, establishing a systematic process for AI model documentation can aid in decoding the intricate internal functions of such models, thereby boosting their credibility [228].

Complexity: For a complex process with dynamic and stochastic effects, multi-scale effects, and many influencing variables, implementing AI will be a challenge. Multiscale modeling to integrate models at different scales to develop a holistic understanding of manufacturing systems for their predictive capabilities comes with huge complexity. Peng et al. [225] explained several challenges regarding the integration of multiscale modeling and machine learning. Exponential increases in complexity can arise due to the complex methodologies used in machine-learning and multiscale modeling. Computationally intensive multiscale modeling and machine-learning models are a challenge to manage in terms of computational resources efficiently. The authors also mentioned that linking multiple scales in a dynamic rather than in a sequential manner is a challenge due to the requirement of consistent communication between different scales.

Strategies that bridge various scales, meticulously adapted to handle dynamic inter-scale interactions, can tackle the intricacies pertaining to multiscale modeling and machine learning, as identified in reference [225]. It is further suggested that a synthesis of data-driven and physics-oriented models can effectively mitigate the complexities inherent in systems associated with nanomanufacturing, as recommended by the study in reference [229].

Security and privacy: All the AI models, like any other computer models, are vulnerable to attacks that generate adversarial predictions [33]. Nano-security is a national priority program for nano-electronics, funded by the German Research Council (DFG), and shows the challenge of vulnerability [230]. Oseni et al. [231] highlighted the importance of security and privacy in AI models and clearly explained the issues of integrity, confidentiality, authenticity, data poisoning, privacy, trojan attacks, and fairness and bias, referencing examples. Some of the challenges mentioned include the following: inputs created with intentional but subtle changes to cause incorrect output, susceptibility to spoofing attacks, minor adversarial instances inserted into the training set causing a model to learn wrong features that erode the performance of the AI models, ML models can sometimes infer sensitive attributes that might compromise individual privacy, trojan attacks by inserting hidden malicious function into the model during the training phase and replicate and amplify the social biases in the training data sets, and inadequate laws and regulations for new technologies. Authors Solanas and Martinez also mentioned similar security and privacy challenges [232]. In their paper, the following were mentioned: improperly anonymized or protected information could be maliciously exploited, a bias in the data may lead to discriminatory practices, cyber-attacks in the manipulation of AI systems, and misuse of AI for nefarious purposes such as deepfake creation. Curzon et al. [233] also agreed to the existence of all the above-mentioned security and privacy issues.

Effective utilization of comprehensive cybersecurity protocols, encryption, and data anonymization techniques can significantly minimize potential security risks. Initiatives to raise awareness and enact appropriate legal regulations are paramount in ensuring security and privacy within AI models. The implementation of deep-learning models has been shown to be a potent tool in the detection and mitigation of adversarial data poisoning in several studies [232]. Additionally, continual monitoring and model updates for the purpose of detecting anomalies or attacks have proven to be helpful in preventing security breaches [233].

Training and implementation: AI systems require extensive training to function efficiently. In nanomanufacturing, where the processes are highly intricate, extensive training would be required. Moreover, changes, evolutions, or alterations to manufacturing processes would require additional system training. Many AI technologies, notably neural networks, are frequently perceived as ‘black boxes.’ This metaphor conveys the complexity and opaque nature of their decision-making processes, making comprehension challenging for many. Implementation of AI involves understanding AI models, their interactions, capabilities, and limitations. The lack of understanding and technical knowledge among professionals could result in resistance to adoption [234]. It was also mentioned that the higher costs to the administrators associated with training AI systems became a challenge in implementing AI.

Addressing the challenge of training and implementation necessitates increased investment in AI education across all levels to enhance the comprehension and acceptance of AI technologies [234]. Herein, regulatory bodies could have a pivotal role by providing incentives to stimulate relevant training programs. Additionally, cultivating a corporate culture that respects and understands AI, and nurturing collaborative relationships between academic institutions and industries, could prove to be mutually beneficial.

Technical issues: AI’s abilities are limited to doing what it has been trained for, and it may be challenging to implement it in settings like nanomanufacturing, where several tasks may need real-time decisions and unexpected problem-solving. Managing and allocating computational resources to process information and make decisions within a given time frame is a key challenge in real-time, according to Musliner et al. [235]. It was mentioned that problems also arise related to task scheduling. In real-time, is difficult to determine the precedence, order, and priority of tasks. Environmental changes or unforeseen circumstances may pose uncertainty and make AI predictions a significant challenge. Apart from the above-mentioned challenges, author Seguin et al. [229] highlighted some more challenges, as follows: the computational time to provide a solution within the required timeframe, computationally expensive solutions under changing conditions, a trade-off between the quality of the solution and the response time, and the requirement of substantial memory space.

The application of real-time machine-learning algorithms, supported by potent computational resources, can provide effective solutions to the challenges of real-time decision-making [235]. Furthermore, the construction of sturdy scheduling algorithms can enhance task scheduling within the nanomanufacturing industry.

Regulations: There are strict regulations in the nanomanufacturing industry to ensure safety and quality standards, especially in the food industry [222]. Fitting AI into these regulations can be a challenge, especially given the dynamic nature of AI and its evolving interpretation of rules [236]. Author Hoffmann raises several challenges regarding law and regulation. One of the major challenges mentioned is the ambiguity and complexity associated with AI, making it difficult to attribute liability due to harm or damage. With AI working autonomously, accountability becomes complex and unidentified. The diversity of AI applications causes regulatory challenges, making it difficult to create comprehensive rules with wider areas of application. With rapid advancements in AI technologies, it is difficult for the law to keep up. AI can have reasonable impacts on society that may go beyond legal considerations, including implications for ethics and human rights, for which careful thought is required regarding law and policy. 

Addressing the complexities of regulatory hurdles necessitates active participation and collaboration from all interested parties, notably developers and regulatory bodies. It is crucial for policymakers to be not only proactive but also adaptable in devising regulatory frameworks. They are expected to adequately predict potential developments, regulate with foresight, and modify policies as required [236].

Equipment compatibility: Implementing AI might require new equipment or might not be compatible with existing machinery or systems, which means companies need to incur a high cost to update their existing systems. Sharma et al. [237] mentioned challenges due to infrastructure. They include a lack of infrastructure, such as high-speed internet, digital devices, or software, and a lack of understanding in identifying the equipment needed to perform the task. Emerging economies might lack the equipment to support AI implementation, and maintenance will be difficult and costly, even with equipment in place.

Addressing equipment compatibility issues necessitates the development of cost-effective tools and methodologies for seamless integration of AI with pre-existing technologies [237]. It is imperative to conduct research focused on the creation of economical and trustworthy AI-compatible machinery. Such advancements can significantly diminish the entry barrier for nano-manufacturers, most notably within developing economies.

Flexibility in material properties: The inherent variability in the properties of nanoscale materials complicates the task of developing a reliable and generalized AI model for nanomanufacturing [238]. At a tiny nanoscale, nanomaterials can exhibit significantly different properties compared with their bulk counterparts. Nanoscale materials and devices can be sensitive to their environment, and their properties can change over time. Training an AI model with dynamics poses a challenge. Even with the same manufacturing conditions, the properties of nanoscale materials can be variable due to the inherent uncertainties in the properties of nanoscale materials. 

Researchers have suggested the creation of comprehensive artificial intelligence models, fundamentally embedded with the intrinsic uncertainties associated with nanoscale properties. They posit that these AI models can be equipped to conduct robust analyses by accommodating the imprecisions and inconsistencies inherently present in the properties of nanoscale materials [238].

Higher costs: Developing and implementing AI algorithms for nanomanufacturing is expensive. It requires investment in extensive data collection, processing, and advanced computing systems, not to mention the expense of hiring experts in both AI and nanomanufacturing to manage the process [239]. The paper by Davenport et al. [239] discusses the economic implications of integrating AI into operations, which include cost challenges. One of the major challenges is the initial investment required for implementation because it involves big data infrastructure, software/hardware, and expert AI practitioners. Even after successful implementation, funds are required for ongoing maintenance and upgrades. 

Recognizing regions of strategic investment and prospective cost savings over an extended period is crucial in counterbalancing implementation expenses. The development of machine-learning algorithms, intended to enhance operational efficiencies, can play a significant role in curtailing operational costs. Moreover, engaging in public–private partnerships could proffer an effective method of distributing the risks and expenses affiliated with the integration of AI technologies. This approach can potentially expedite adoption and contrive a cost-effective strategy for implementing these tech-driven solutions.

### 4.2. Discussion and Future Prospects

Bridging nanomanufacturing and artificial intelligence (AI) involves integrating AI technologies into the processes of designing, fabricating, and characterizing nanoscale materials and devices. This review paper highlights the transformative potential of integrating AI technologies into nanoscale manufacturing processes. This integration has the potential to revolutionize the field of nanomanufacturing by enabling advanced capabilities in material discovery, process optimization, quality control, and predictive modeling. In this paper, some key aspects we considered are: 

Nanomanufacturing and Industry 4.0: This paper introduces nanomanufacturing as a critical component of Industry 4.0, emphasizing its role in producing materials and devices at the nanoscale. It discusses how nanomanufacturing enables the creation of innovative products with unique properties and functionalities, setting the stage for the integration of advanced technologies like AI.

Role of AI in Nanomanufacturing: This paper explores the diverse applications of AI in nanomanufacturing, highlighting its significance in predicting material properties, optimizing manufacturing processes, data-driven decision support, and enhancing operational efficiency. The prospects of AI in nanomanufacturing encompass accelerated material discovery, customized nanomaterial design, advanced nanodevices, and efficient resource utilization, paving the way for transformative advancements in nanotechnology and advanced manufacturing. AI algorithms are portrayed as powerful tools for real-time monitoring, quality control, and predictive maintenance in nanomanufacturing operations [220,221]. 

Material Discovery and Exploration: AI’s role in material discovery and exploration within nanomanufacturing is discussed in detail. By analyzing vast datasets and identifying patterns, AI algorithms facilitate the discovery of new materials with unique characteristics, potentially leading to breakthroughs in diverse fields such as nanoelectronics, photonics, and biomedicine. This capability drives innovation in product development and design, enabling companies to stay at the forefront of technological advancements. The prospects include the rapid development of materials for emerging technologies, the creation of novel nanomaterial libraries, and the integration of diverse expertise for breakthrough innovations. These prospects contribute to the continual advancement of nanotechnology and its diverse applications across various industries and scientific domains [199,200,201]. 

Process Optimization and Efficiency: This paper delves into how AI-driven process optimization enhances efficiency and productivity in nanomanufacturing. AI algorithms analyze data in real-time, recommend adjustments, and minimize wastage, leading to improved operational efficiency and reduced defects. The automation and streamlining of processes through AI technologies contribute to overall process optimization. These prospects contribute to the continual advancement and widespread adoption of nanotechnology in various industrial sectors, driving economic and technological progress.

Quality Control and Predictive Maintenance: AI’s critical role in quality control and predictive maintenance is emphasized. AI-powered quality control mechanisms ensure consistent product quality and minimize defects, while predictive maintenance strategies optimize maintenance schedules and prevent equipment failures. This involves the utilization of advanced characterization techniques such as electron microscopy, spectroscopy, and atomic force microscopy to assess the structural, chemical, and morphological properties of nanomaterials and products. This ensures adherence to stringent quality standards and specifications. This proactive approach to maintenance enhances operational efficiency and ensures continuous operation of manufacturing systems [222,223]. 

Innovation, Research Advancements, and Competitive Edge: AI fosters innovation, drives research advancements, and enhances competitiveness in nanomanufacturing. By applying AI technologies, companies gain a competitive edge by improving efficiency, product quality, and market relevance. AI-driven innovation enables companies to differentiate themselves, optimize operations, and meet evolving customer demands. Future research directions may focus on further refining AI algorithms for nanomanufacturing applications, addressing challenges such as data availability, security, and cost to unlock the full potential of AI in driving innovation and competitiveness.

Market Differentiation and Long-Term Sustainability: The integration of AI into nanomanufacturing not only provides immediate benefits in terms of efficiency and quality but also ensures long-term sustainability and competitiveness. Companies that invest in AI technologies for nanomanufacturing are better positioned to innovate, adapt to market changes, and sustain their competitive edge over time. By offering customized nanoproducts, embracing sustainable practices, and addressing ethical considerations, nanomanufacturers can position themselves for long-term success, growth, and positive industry impact. 

Production systems are becoming more sophisticated, dynamic, and interconnected, causing nonlinear and stochastic activity issues. AI, particularly machine learning, has the potential to transform manufacturing by processing large volumes of industrial data, known as big data [240]. However, gaps must be addressed before AI can be integrated into nanomanufacturing operations.

Optimum performance necessitates a substantive understanding of the intricacies involved in the relationships between material processing and property. Development and implementation of conventional modeling and control mechanisms can be integrated into the process to address the change in material properties during the process and to solve the operational issues on a real-time basis [241]. For a different challenge, Malaca et al. [242] proposed computer vision-based AI models be developed to match the variable process operating conditions. The same concept is also proposed by Guven et al. [243] to address ever-changing laboratory operations. The intricate and unpredictable dynamics of manufacturing systems necessitate a comprehensive understanding of the multifaceted processes involved. The vast quantities of generated data, diverse in their characteristics and dependent on numerous variables, require the application of suitable algorithms to aid in the effective analysis and interpretation of these complex systems [196,240]. Machine learning with image recognition, such as microscopy, can generate more data, which is essential to getting high accuracy levels for an ML algorithm. Since manufacturing operations are based on physics, not on probability theories as used in AI, more transparent, physics-guided process models such as explainable AI (XAI), which supports mental support models [240], can be studied further. With very limited research conducted on the biological effects of nanoplastics, researchers can use ML algorithms to explore the same as a part of artificial intelligence [197].

The implementation of AI in nanomanufacturing presents several challenges, along with emerging solutions and prospects. Some of the main challenges include issues with data availability and quality, the understandability of AI and ML results, complexity in multiscale modeling, issues with security and privacy, training hurdles, technical issues in real-time decision-making, stringent regulations, equipment compatibility, and costs, as well as variability in material properties. However, recent developments in AI show the potential to transform the manufacturing domain with the use of advanced analytic tools. Potential solutions proposed by researchers include the development of conventional modeling and control mechanisms, computer vision-based AI models, reinforced learning algorithms, and physics-guided models like explainable AI. The exploration of these new solutions could mitigate the challenges and pave the way for seamless integration.

## 5. Conclusions

This paper fills a significant gap in the literature by providing a comprehensive review of the integration of artificial intelligence in nanomanufacturing processes. It focuses on how AI can optimize material synthesis, process control, and quality assurance in nanomanufacturing. Furthermore, it reveals the potential of AI in enhancing competitiveness and market relevance by improving product quality, reduce costs, and accelerate time-to-market, hence offering strategic insights for organizations seeking to expand and enhance their manufacturing capabilities. Another key justification for this review is that it discusses how AI resonates with the principles of Industry 4.0, emphasizing digitalization, automation, and data-driven decision-making in nanomanufacturing. By fostering cross-disciplinary collaboration and knowledge exchange between experts in materials science, engineering, and AI, these efforts can lead to breakthroughs in nanomanufacturing technology. In addition, by identifying key challenges, opportunities, and emerging trends, this paper serves as a guide for further exploration and innovation in this rapidly evolving area.

The scientific paradigm underlying materials at the nanoscale, developments in nanomanufacturing, digital fabrication, and the advent of emerging artificial intelligence frameworks represent the frontier of the fourth industrial revolution. In this review, we discuss the integration of artificial intelligence in nanomaterial exploration, manufacturing, and metrology, offering exciting opportunities for optimization in synthesis, processing, and characterization. Different AI algorithms that include supervised, unsupervised, reinforcement, and deep learning are discussed in the context of nanomaterial discovery, property enhancement, and nanomanufacturing predictive analytics. Moreover, significant challenges need to be overcome for the implementation of AI, such as data availability, security, high costs, lack of expertise, and legal and ethical issues. Hybrid models combining AI with other heuristic algorithms can pave the way for real-time control and feedback of nanomanufacturing processes. A notable development is the application of reinforced learning concepts that could significantly increase productivity levels. Furthermore, the advent of explainable AI (XAI) is a step towards creating a more reliable and user-friendly digital platform as a physics-guided process model to make the AI’s decision-making process understandable for its users. It ensures a transparent interplay between data analysis and physical constraints, to provide judicious guidance for material designers and manufacturers. Future research is needed to fully integrate and exploit AI algorithms to further revolutionize material synthesis and the manufacturing industry, paving the way for next-generation solutions.

## Figures and Tables

**Table 1:** Nanomanufacturing and digital manufacturing relationship.

Context	Relation between Nanomanufacturing (NM) and Digital Manufacturing (DM)
Industry 4.0	Industry 4.0 is facilitated by digitalization and integration technologies to build cyber-physical systems and smart factories [31]. Almakaeel et al., discussed extensively the combination of digital concepts with several artificial neural networks to predict the optimal nano/micro-manufacturing process [33].
Computer systems	NM and DM both use computer systems to design, simulate, analyze, and produce a product.
Requirement	NM and DM require a high level of research and development and require highly advanced equipment and technology. In addition, a highly skilled workforce to operate and manage the technology is required.
Control	In most cases, nanomanufacturing processes are controlled using digital manufacturing techniques.
End goal	Both NM and DM offer opportunities for greater efficiency, cost savings, and customization in production.
Future	The integration of NM and DM could lead to the development of newer materials and processes, thereby enhancing the production capabilities.

**Table 2:** Nanomanufacturing with the role of AI.

Nanomanufacturing Process	Role of AI	Ref.
Nano assembly: use of dielectrophoresis (DEP), the utilization of electrokinetic force employed during the assembly at the nanoscale.	Automated ascertainment of frequency range for positive and negative dielectrophoresis.	[52]
Carbon nanotubes and graphene were used in the preparation of grease as additives to improve the grease properties. Microwave-assisted ball milling was the process.	The performance of the grease was continuously monitored using an artificial neural network (ANN)	[53]
Nanomedicine where nanometric structures provide a highly sophisticated, targeted approach to diagnosing health conditions.	AI is used to optimize the properties of materials relative to anticipated interactions with the designated medication to prepare the cancer medicine.	[54]
Nanorobotics, characterized by their nanoscale sizes, are employed in the field of biomedical applications.	Artificial intelligence and biomedicine are used in creating nanorobots.	[55,56]
Nanocomposite multifunctional mechanical wearable sensors.	AI is used to predict the outcomes for parameters such as breathing, heart rate, cardiovascular system signals, and pressure in joint surgeries.	[56,57,58,59,60]
Luminescence sensors manufacturing with nanomaterials.	Machine-learning (ML) models have significantly contributed to the improvement of efficiency, sensitivity, and selectivity within luminescence platforms.	[61]

**Table 3:** Machine-learning algorithms used in the synthesis of nanoparticles.

Nanoparticles	Description	Machine-Learning Models	Ref.
Gold nanoparticles	ML models were utilized to analyze the correlation between nanoparticle size, quantity of reagents, and bacteria strain effects on nanoparticles.	LASSO regression, RR, ENR, SVM, RF	[86]
NiO and TiO	Employed ANN to demonstrate effective techniques for utilizing ultra-low power inert gas condensation (ULPING) to create nanoscale structures.	ANN	[87]
Gold nanoparticles	Utilized ANN to understand the correlation between the ratio of sodium citrate to gold salt for gold nanoparticle generation.	ANN	[88]
Silver nanoparticles	Two ML (BO and DNN) were utilized to improve the Accuracy of model predictions for optimizing the synthesis of silver nanoparticles.	BO, DNN	[89]
CsPbBr3 type perovskite	Several models, such as SVM regression, linear regression, and quadratic regression, were developed to control the synthesis of perovskite halide nanoplatelets.	SVM	[90]
Colloidal quantum dots	ML was used to speed up the synthesis of colloidal quantum dots.	ANN	[91]
Perovskite oxides	ML analysis of perovskite oxides grown by molecular beam epitaxy.	K means	[92]
CNT forest	Using ML to predict carbon nanotube forest attributes	DL, RFF	[93]
SrRuO_3_	Utilized BO in molecular beam epitaxy	BO	[94]
CsPbBr3	Utilized active learning algorithm to determine the optimal synthesis route	Active learning algorithm	[95]

**Table 4:** Studies use ML models to predict the properties of polymeric nanocomposites.

Nanocomposite	Description	ML Model	Ref.
Polymer/SiO_2_	ANN and ANFIS were developed to predict the fracture energy of polymer nanocomposites using data from various experimental datasets. Results indicated it was proportional to the experimental data.	ANN + ANFIS	[105]
Epoxy/SiO_2_	ANN was utilized to predict the fracture toughness of particulate polymer composites. The inputs found important, in this order, were time > aspect ratio > elastic modulus > volume fraction. Results indicated that rod-shaped fillers exhibited enhanced fracture toughness.	ANN	[106]
PTFE/CF/TiO_2_	ANN was utilized to predict the tribology properties of short-fiber composite.	ANN	[107]
PLA/GNP	Development of a back-propagation ANN to predict the mechanical property of SPS-processed GNP/PLA polymer nanocomposites. Predictions were proportional to experimental test results.	ANN	[108]
Polymer/nanofiller	A deep multi-task convolutional network was utilized to analyze polymer nanocomposite structure–property relationships and to predict its mechanical properties.	CNN	[109]
PPy/MWCNT	Novel MWCNTs-PPy-embedded RO nanocomposite membranes were synthesized then ANN was utilized for the prediction of model flux measurements.	ANN	[110]
PA-6/Nanoclay	ANN was utilized as a predictive model for optimizing surface roughness in Polyamide-6 nanocomposites based on milling parameters and nanoclay content. GA was utilized to obtain optimized machining conditions.	ANN + GA	[111]
Polymer/CNT	ANN was developed to predict the behavior pattern of the Young’s modulus of polymer/CNTs composites.	ANN	[112]
Polymer/GNP	Proposed neural networks to describe the nonlinear electric conduction in random graphene-polymer nanocomposites.	ANN	[113]
Starch/Clay/AgNps	ANN with LM feed forward was utilized for optimization, evaluation and prediction analysis, and modeling of chemical reactions (AGNP size in the bio-nanocomposite matrix).	ANN + LM	[114]
CNT/Epoxy	SVR with PSO were employed to develop a model to predict the mechanical properties of CNT/epoxy composites. It successfully achieved high accuracy and consistency with experimental results.	SVR + PSO	[115]
Clay/Silica	ML models were used to predict the mechanical properties of a polymer clay nanocomposite before its synthesis. Characterization was also performed by XRD and SEM.	ANN	[116]

**Table 5:** AI algorithms in the exploration of nanomaterials.

Area of Application	AI Algorithm(s)	Purpose	Reference(s)
Discovery of new nanomaterials	Recurrent neural networks (RNNs) and Monte Carlo tree search.	Utilized in predicting the growth of clusters into nanoparticles and recovery of structures in thin films.	[199,200,201]
Nanoscale structure descriptors	Multichannel convolutional neural network (CNN).	Utilized in predicting the cytotoxicity of virtual carbon nanoparticles, predicting disease-prone protein binding sites, and nanoparticle material properties.	[202,203,204,205]
Defect analysis (surface and internal structural)	Linear regression (LR), random forest (RF), and deep learning.	To detect the defects in nanofibrous materials, microencapsulated materials, lattice defects, and polymer composites.	[206,207]
Exploring phase-changing materials (PCMs) and thermoelectric effect materials	Digital tree, elastic net (EN) quadratic polynomial LASSO (QP-LASSO), and neural networks.	To explore highly active catalysts for energy conservation.	[208]
Optical properties of the materials	Deep learning with bi-directional neural networks.	To predict the refractive index of organic polymers.	[209,210]
Thermal properties of the materials	Linear regression, polynomial regression, decision trees, random forest, and artificial neural networks (ANN).	To predict interfacial thermal resistance thermal interface materials.	[211]
Electronic properties of materials	Multiple linear regression (MLR), DT, KNN, ANN, and SVM models.	Utilized in predicting electronic properties and energy differences in graphene nanoflakes.	[212,213]
Exploring nanomaterials with high adsorption capacity	Deep neural networks	Utilized in predicting the adsorption of organic pollutants.	[214]
To study the interaction between nanomaterials and biology	Random forest (RF)	To predict the formation and composition of protein coronas.	[68,215]

**Table 6:** Commonly used AI algorithms.

Type of Algorithm	Algorithm Name	Description
Supervised Learning [216]	Linear Regression	Linear regression models are constructed with input variables relevant to the output variable and they are not highly correlated, used for regression problems.
	Logistic Regression	Logistic regression is for the numeric input variables to model binary classification problems, used for classification problems.
	Random Forest	In a random forest model, instances from the training dataset are selected, utilizing a replacement methodology. Further, the development of trees is strategically performed to mitigate the correlation observed among individual classifiers, thus enhancing the efficacy and reliability of the model.
	Gradient Boosting	Gradient boosting is constructed for classification using the gradient boosting classifier class.
	Support Vector Machines (SVM)	SVM is prepared with a line that separates the two classes. The data closest to the line are called support vectors, used for multiple classes.
	Decision Trees	Decision trees construct a binary tree from the data and split the data to minimize a cost function.
	Naïve Bayes	Naïve Bayes calculates the probability of each class based on each input value.
	Neural Networks	Neural networks are predominantly composed of intricately interconnected nodes, also known as neurons, which are systematically organized into various layers. Each corresponding connection within the network is affiliated with a specific weight. This said weight is duly adjusted over the course of the training process to decrease the error rate to the lowest possible extent.
Unsupervised Learning [217]	Principal Component Analysis (PCA)	PCA uses linear algebra to convert the dataset into a compressed form to reduce the data, called feature extraction.
	K-Means Clustering	In K-means clustering, data are paired with values as clusters.
	Hierarchical Clustering	Establishing a hierarchy of clusters is the main idea behind the hierarchical clustering, to check the data anomalies that go beyond the clusters.
	Apriori	Apriori is for data mining, to extract data from a large pool of data with various transactions.
	ECLAT	Equivalence class clustering and bottom-up lattice traversal (ECLAT) is also for data mining, to locate frequent items.
Reinforcement Learning [218]	Q-Learning	Q-learning finds the optimal solution in a Markov decision process by maintaining a Q-table with the combination of states and actions.
	State-Action-Reward-State-Action (SARSA)	In the SARSA algorithm, the current state action is updated based on the next action.
	Deep Q Network	The deep Q network works with discrete actions. It combines convolutional and pooling layers, followed by fully connected layers.
	Monte Carlo Methods	The Monte-Carlo algorithm works with episodic cases and considers complete episodes to obtain gradient values.
Deep Learning [219]	Conventional Neural Networks (CNN)	CNNs are applied for object recognition, detecting digits, faces, and objects with different orientations. Features are learned and used across the whole image.
	Recurrent Neural Networks (RNN)	RNNs are designed for sequence problems. Additional loops are used in the network in addition to the feedforward multilayer perceptron network.
	Long Short-Term Memory Networks (LSTM)	LSTM is an RNN, trained using backpropagation. These networks use memory blocks that are connected into layers.
	Autoencoders (AE)	Encoders work by encoding the strings to integers, then convert the vector of integers to a one-hot encoding.

## Data Availability

The data presented in this study are available on request from the correspondence author.
